# Atypical Presentation of Median Arcuate Ligament Syndrome in the Emergency Department

**DOI:** 10.5811/cpcem.2019.9.44075

**Published:** 2019-10-21

**Authors:** Abby Sapadin, Ryan Misek

**Affiliations:** Midwestern University Chicago College of Osteopathic Medicine, Department of Clinical Education, Downers Grove, Illinois

## Abstract

Celiac artery compression syndrome, also called median arcuate ligament syndrome (MALS), is a rare condition in which the diaphragmatic crura compresses the celiac axis. This results in a constellation of primarily gastrointestinal (GI) symptoms including nausea, vomiting, postprandial abdominal pain, and weight loss. It is typically a diagnosis of exclusion and may be detected via several imaging techniques including ultrasound and computed tomography angiography. We present an atypical case of MALS detected in the emergency department (ED). We review the symptomatology, diagnostic workup, and treatment options here, as well as discuss implications concerning revisits to the ED for recurrent GI symptoms.

## INTRODUCTION

Abdominal pain is one of the most common reasons patients present to the emergency department (ED) and carries a myriad of possible underlying pathologies. While it is a relatively benign condition, median arcuate ligament syndrome (MALS) may mimic life-threatening causes of abdominal pain. This case describes this patient’s unique presentation, reviews current diagnostic and treatment modalities for this condition, and discusses its place in the broader context of revisits to the ED.

## CASE REPORT

A 59-year-old male presented to the ED with complaints of crampy, diffuse abdominal pain with associated nausea and watery diarrhea ongoing for the prior four days. He had been evaluated in the ED two days prior to presentation with similar complaints, at which time his workup was significant only for diverticulosis on computed tomography (CT) of the abdomen and pelvis with intravenous contrast. He had been discharged home in stable condition with oral dicyclomine 10 milligrams (mg) and ibuprofen 600 mg. The patient followed up with his primary care physician the next day, at which time he was prescribed hydrocodone/acetaminophen 5/325 mg for pain control. Due to progressively worsening abdominal pain not relieved with these medications, he returned the ED for further evaluation.

The patient’s past medical history was significant for Barrett’s esophagus, chronic kidney disease, diverticulosis, gastroesophageal reflux disease, gout, hyperlipidemia, and hypertension. He had undergone esophagogastroduodenoscopy (EGD) for GI complaints in the past. He was compliant with his home amlodipine, atorvastatin, lisinopril, and omeprazole. Social history was significant for a 45-pack per year smoking history and weekly, moderate alcohol consumption.

Upon arrival to the ED, the patient’s vitals revealed a temperature of 97.9 degrees Fahrenheit, pulse 71 beats per minute, respiratory rate 20 breaths per minute, blood pressure 150/89 millimeters of mercury (mmHg), and pulse oximetry 98% on room air. Physical examination revealed a nontoxic patient, mildly uncomfortable appearing, but in no acute distress. He did have mild, diffuse abdominal tenderness to palpation without distention, rebound, or abdominal bruit. The remainder of his physical examination was unremarkable. Initial diagnostic workup including complete blood count, complete metabolic panel, lipase, urinalysis, urine drug screen, lactate, electrocardiogram, troponin I, and chest radiograph was unremarkable.

**Image 1:**
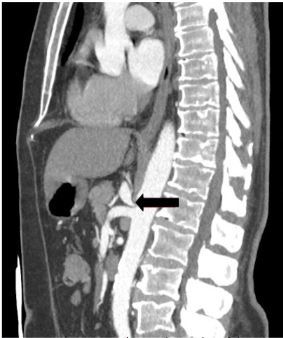
Computed tomography angiography of the abdomen and pelvis sagittal view demonstrating characteristic “hooked” appearance (arrow) of celiac artery, origin off the abdominal aorta.

**Image 2:**
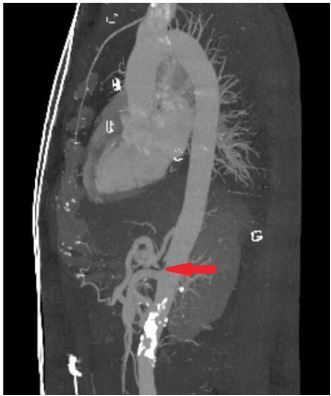
Three-dimensional reconstruction of computed tomography angiography of the abdomen and pelvis sagittal view demonstrating characteristic “hooked” appearance of celiac artery (arrow), origin off the abdominal aorta.

Upon reevaluation, the patient’s symptoms had somewhat improved, but he then reported some tenderness to palpation in the midline of his back. A CT angiogram of the chest and abdomen with three-dimensional reconstructions was obtained and revealed stenosis at the origin of the celiac artery with characteristic “hooked” appearance, raising concern for MALS (Images 1 and 2). The patient was then reassessed and reported spontaneous relief of his symptoms. He was informed of his imaging findings and discharged home in stable condition with referral to general surgery to explore possible treatment options. He subsequently followed up with general surgery and was referred to the interventional radiology (IR) service at a tertiary care center.

Prior to recommending IR angioplasty and stenting, the patient underwent EGD, colonoscopy, and cholescintigraphy, which were normal. The patient then had an abnormal stress test as part of cardiac clearance for surgery, leading to subsequent cardiac catheterization and percutaneous coronary intervention. At follow-up with his primary care physician 10 months after the initial ED visit, the patient was still exhibiting similar GI symptoms and was awaiting cardiology clearance to proceed with IR angioplasty and stenting.

## DISCUSSION

MALS, also called celiac artery compression syndrome or Dunbar syndrome, is a rare condition in which low-lying fibers from diaphragmatic crura compress the celiac artery or ganglion.^1^ Typically, the ligament lies anterior to the aortic hiatus, uniting fibers from either side of the diaphragmatic crura superior to the celiac axis. However, in up to 24% of the population, the ligament crosses anterior to the celiac artery and in some individuals may compromise blood flow, leading to concurrent symptomatology.^2^ The condition was first described in 1963 by Harjola in a case report in which a patient presented with epigastric bruit and postprandial abdominal pain and was found to have narrowing at the celiac axis secondary to local fibrotic tissue.^3^ Compression or stenosis in this area is thought to cause postprandial foregut ischemia, resulting in a constellation of GI symptoms including postprandial epigastric pain, nausea, vomiting, diarrhea, bloating, and unintentional weight loss secondary to food aversion.^1^–^4^ Physical examination may also reveal an audible abdominal bruit best heard over the epigastrium, which is detected in up to 83% of cases.^5^,^6^

**CPC-EM Capsule**

What do we already know about this clinical entity?

*Median arcuate ligament syndrome (MALS) is a rare condition that typically produces chronic gastrointestinal (GI) symptoms*.

What makes this presentation of disease reportable?

*This case was detected via imaging in the emergency department (ED) rather than the outpatient setting. Additionally, this patient did not fit the typical demographics for this condition*.

What is the major learning point?

*MALS may be detected in the ED. Patients with imaging suspicious for this condition will require GI and surgical referral for further diagnostic and therapeutic management*.

How might this improve emergency medicine practice?

*Readers will include MALS in their differential for patients with multiple ED visits for GI complaints, and will know appropriate disposition if this condition is suspected*.

While celiac axis compression and subsequent foregut ischemia is the most widely accepted understanding of MALS pathophysiology, this is debatable as multiple studies have suggested the symptoms are secondary to irritation of the celiac ganglion as opposed to intestinal ischemia.^7^,^8^ A significant portion of the general population may have some degree of median arcuate ligament compression without symptomatology, with estimates varying from 3.42% in a study of asymptomatic patients with compression noted on CT to 10–24% in a comprehensive literature review of MALS cases.^4^,^9^

Conversely, the incidence of MALS is estimated at approximately two per every 100,000 patients.^10^ The majority of MALS patients are relatively young women between the ages of 20–50 years old with thin body habitus who have been extensively worked up for various intra-abdominal pathology.^1^,^6^ As MALS is a diagnosis of exclusion, initial gastroenterology referral may be required if patients have not had prior extensive workup to investigate other possible causes of functional abdominal pain. Once other causes have been ruled out, the diagnosis can be confirmed with imaging including duplex ultrasound, CT angiography, and magnetic resonance angiography revealing compression or stenosis of the celiac artery.^6^,^8^ Percutaneous diagnostic celiac ganglion blockade, in which lidocaine is injected into splanchnic nerves feeding into the celiac plexus, can also be performed by general surgery or IR to predict symptomatic improvement with surgical intervention.^10^–^12^

Treatment historically consists of surgical techniques, although due to the rarity of the syndrome there is no single accepted treatment algorithm and options should be considered in the context of an individual patient’s age and severity of symptoms. General surgeons may perform minimally invasive techniques such as laparoscopic or robotic median arcuate ligament release, while vascular surgeons may perform percutaneous transluminal angioplasty, primary reanastomosis, or celiac artery bypass grafting in refractory cases.^6^,^13^–^17^ IR may also perform angioplasty with stenting.^14^,^18^ The success rate of immediate postoperative symptom relief has been reported at up to 85%, with sustained symptom relief for years following surgery reported between 53–80% with surgical decompression alone and over 80% relief with both decompression and other vascular intervention.^19^,^20^ Patients should thus be counseled that multiple interventions may be necessary for the best chance of long-term symptom relief.

The presentation of MALS discussed in this case report is unusual in that it was in a moderately obese, slightly older male. His physical examination findings were nonspecific, and his history lacked the characteristic postprandial food aversion seen in many MALS cases. Furthermore, this condition is primarily detected in the outpatient setting following years of chronic GI symptoms as opposed to acutely in the ED. While MALS is not life-threatening, it may mimic the symptoms of mesenteric ischemia and may initially be worked up similarly in the ED, as was done in this case.^21^ Therefore, this diagnosis should be considered in any patients presenting to the ED with recurrent GI complaints with previously negative workups. If MALS is suspected based on imaging obtained in the ED, providers should refer patients to gastroenterology for further workup and exclusion of other causes of chronic abdominal pain, with general or vascular surgery referrals if the patient records demonstrate a history of extensive workup for GI symptoms.

## CONCLUSION

Median arcuate ligament syndrome is a rare cause of recurrent abdominal pain and is typically a diagnosis of exclusion. While a significant portion of the United States population may have some underlying compression of the celiac axis, a much smaller percentage of these individuals may develop symptoms of MALS. The above case brings awareness of this uncommon disease to ED professionals, and demonstrates that it may occur in patients who do not fit its typical epidemiological demographics. Its presentation may mimic that of mesenteric ischemia and should be considered in any patient presenting to the ED multiple times for abdominal pain with previously unremarkable workups. Early recognition of atypical vascular causes of abdominal pain with proper outpatient referral may decrease costly ED revisits and help patients with chronic GI complaints ultimately find lasting relief.
